# Three-Dimensional *in vitro* Models of Healthy and Tumor Brain Microvasculature for Drug and Toxicity Screening

**DOI:** 10.3389/ftox.2021.656254

**Published:** 2021-05-04

**Authors:** Marie Piantino, Agathe Figarol, Michiya Matsusaki

**Affiliations:** ^1^Department of Applied Chemistry, Graduate School of Engineering, Osaka University, Osaka, Japan; ^2^Institut Jean Lamour, UMR 7198 CNRS, Université de Lorraine, Nancy, France

**Keywords:** blood-brain barrier, *in vitro* model, vascularization, glioblastoma, toxicity

## Abstract

Tissue vascularization is essential for its oxygenation and the homogenous diffusion of nutrients. Cutting-edge studies are focusing on the vascularization of three-dimensional (3D) *in vitro* models of human tissues. The reproduction of the brain vasculature is particularly challenging as numerous cell types are involved. Moreover, the blood-brain barrier, which acts as a selective filter between the vascular system and the brain, is a complex structure to replicate. Nevertheless, tremendous advances have been made in recent years, and several works have proposed promising 3D *in vitro* models of the brain microvasculature. They incorporate cell co-cultures organized in 3D scaffolds, often consisting of components of the native extracellular matrix (ECM), to obtain a micro-environment similar to the *in vivo* physiological state. These models are particularly useful for studying adverse effects on the healthy brain vasculature. They provide insights into the molecular and cellular events involved in the pathological evolutions of this vasculature, such as those supporting the appearance of brain cancers. Glioblastoma multiform (GBM) is the most common form of brain cancer and one of the most vascularized solid tumors. It is characterized by a high aggressiveness and therapy resistance. Current conventional therapies are unable to prevent the high risk of recurrence of the disease. Most of the new drug candidates fail to pass clinical trials, despite the promising results shown *in vitro*. The conventional *in vitro* models are unable to efficiently reproduce the specific features of GBM tumors. Recent studies have indeed suggested a high heterogeneity of the tumor brain vasculature, with the coexistence of intact and leaky regions resulting from the constant remodeling of the ECM by glioma cells. In this review paper, after summarizing the advances in 3D *in vitro* brain vasculature models, we focus on the latest achievements in vascularized GBM modeling, and the potential applications for both healthy and pathological models as platforms for drug screening and toxicological assays. Particular attention will be paid to discuss the relevance of these models in terms of cell-cell, cell-ECM interactions, vascularization and permeability properties, which are crucial parameters for improving *in vitro* testing accuracy.

## Introduction

### Importance of Vascularization in 3D Engineering

For decades, the construction of vascularized tissue has been a major challenge in 3D tissue engineering for non-animal alternatives. Vascularization is essential to supply thick tissue (>100–200 μm) with sufficient oxygenation to allow their long-term maintenance, and conduct, for example, toxicity assessments in sub-acute and sub-chronic conditions (Chang and Niklason, 2017). It is also especially important for modeling pathological tissues and improving treatments. In particular, drug candidates for neurological pathologies have higher failure rates at the bench-to-bed transition than any other drugs (Gribkoff and Kaczmarek, 2017). It has been demonstrated that of the mere 8% of candidates reaching the initial Phase 1 of human safety testing, only a limited number received commercial approval (DiMasi et al., 2010). The adverse effects on the brain microvasculature are still poorly understood, and the question is whether they arise from endogenous pathological mechanisms or from the drugs themselves. Damage or dysfunction of the brain vasculature are often associated with many neurological diseases, including brain cancers. Glioblastoma multiform (GBM) is not only one of the most common forms of brain cancer in adults, but also one of the deadliest brain tumors, with a median survival of 12 months with appropriate treatments. It is also one of the most vascularized brain cancers, and is associated with a high remodeling of the ECM There has been intensive research dedicated to modeling the characteristic features of GBM in order to understand its impact on brain vascularization, particularly the regulation of the angiogenic signaling pathways, as microvascular proliferation is a hallmark of GBM (Hardee and Zagzag, 2012; Rodriguez et al., 2012).

The shortage of effective therapies and low success rate of investigational drugs are partly due to the lack of reliable human equivalent models (Nzou et al., 2018). Many studies have attempted to model the dynamic and complex structure that represents the brain vasculature for a better understanding of drug permeation through the brain. Traditionally, the most common models are two-dimensional (2D) as they are quite simple and easy to prepare, making them useful for high-throughput drug screening (HTS) (Hatherell et al., 2011). However, 2D *in vitro* models fail to reproduce the BBB's key properties as they do not take into account the 3D cellular organization and the importance of direct cell-cell interactions, which are critical factors for proper cellular differentiation, and the polarization of the cells (Hatherell et al., 2011; Gribkoff and Kaczmarek, 2017). The relevance and related ethical issues arising from animal models also limit their use for the investigation of drug delivery in the brain (Figarol and Matsusaki, 2020). Although there have been rising concerns about the use of animals for drug delivery and toxicological assays, animals continue to be used worldwide for scientific purposes. A recent study by Taylor and Alvarez has estimated the animal testing numbers worldwide, with an increase from 115.2 million animals to 192.1 million between 2005 and 2015 (Taylor and Alvarez, 2019). According to European Union definitions, China and Japan were ranked first and third place for animal uses in 2015, with estimated 20.5 and 5 million procedures, respectively (Taylor and Alvarez, 2019). It seems thus unlikely to see a significant reduction of animal testing in the immediate future. Nevertheless, there have been increasing efforts from the scientific community and pharmaceutical companies to limit the proportion of animal research, whenever possible, though the development of alternative techniques, such as advanced biomimetic *in vitro* cellular models of the brain microvasculature.

### Physiology of the Blood-Brain Interface

The brain is one of the most important organs in the body because it regulates many vital functions such as the processing of information arising from our senses, the control of our thoughts and movement, as well as the regulation of breathing and blood pressure. Exchanges occur at the blood-brain interface to enable sufficient brain nutrition and oxygenation, as well as waste removal (Abbott et al., 2010). In the average human adult brain, the surface exchange area between itself and the vascular system is between 12 and 18 m^2^ (Pardridge, 2007). Blood vessels can be categorized depending on their size and diameter, with large vessels (>6 mm in diameter), small vessels (1–6 mm) and microvessels or capillaries (<1 mm) (Chang and Niklason, 2017). The brain vasculature is a highly complex network which comprises of arteries and arterioles, capillaries, venules, and veins. The large surface exchange area is mainly due to the presence of the brain microvasculature comprising more than 100 billion capillaries, with a density of about 500 m/cm^3^, which correspond to an average length of about 600 km (Pardridge, 2007; Wong et al., 2013). Modeling the brain microvasculature is thus of importance to collect more relevant *in vitro* data simulating the drug permeation or toxicity assessment of compounds in the brain for improved clinical translations.

Because of the dense vascular network in the brain, blood circulation provides a readily accessible route for neuropathic medications, provided that they can cross the blood-brain barrier (BBB). The BBB forms indeed a protective barrier, located at the cerebral capillary endothelial cells. This barrier helps maintain the homeostasis of the central nervous system (CNS) by tightly regulating the entry of molecules, ions and cells, and is essential for proper neuronal function (Chow and Gu, 2015). It is one of the most restrictive biological barrier in the human body, impeding not only xenobiotics but also various undesirable proteins, antibodies, and even small molecules, from entering the brain parenchyma. For example, it has been demonstrated that the BBB blocks the passage of 100% of large molecules (>1,000 Da) and >98% small-sized (<500 Da) drug molecules, which is a challenge for treating neurological pathologies (Pardridge, 2007).

**Figure 1:**
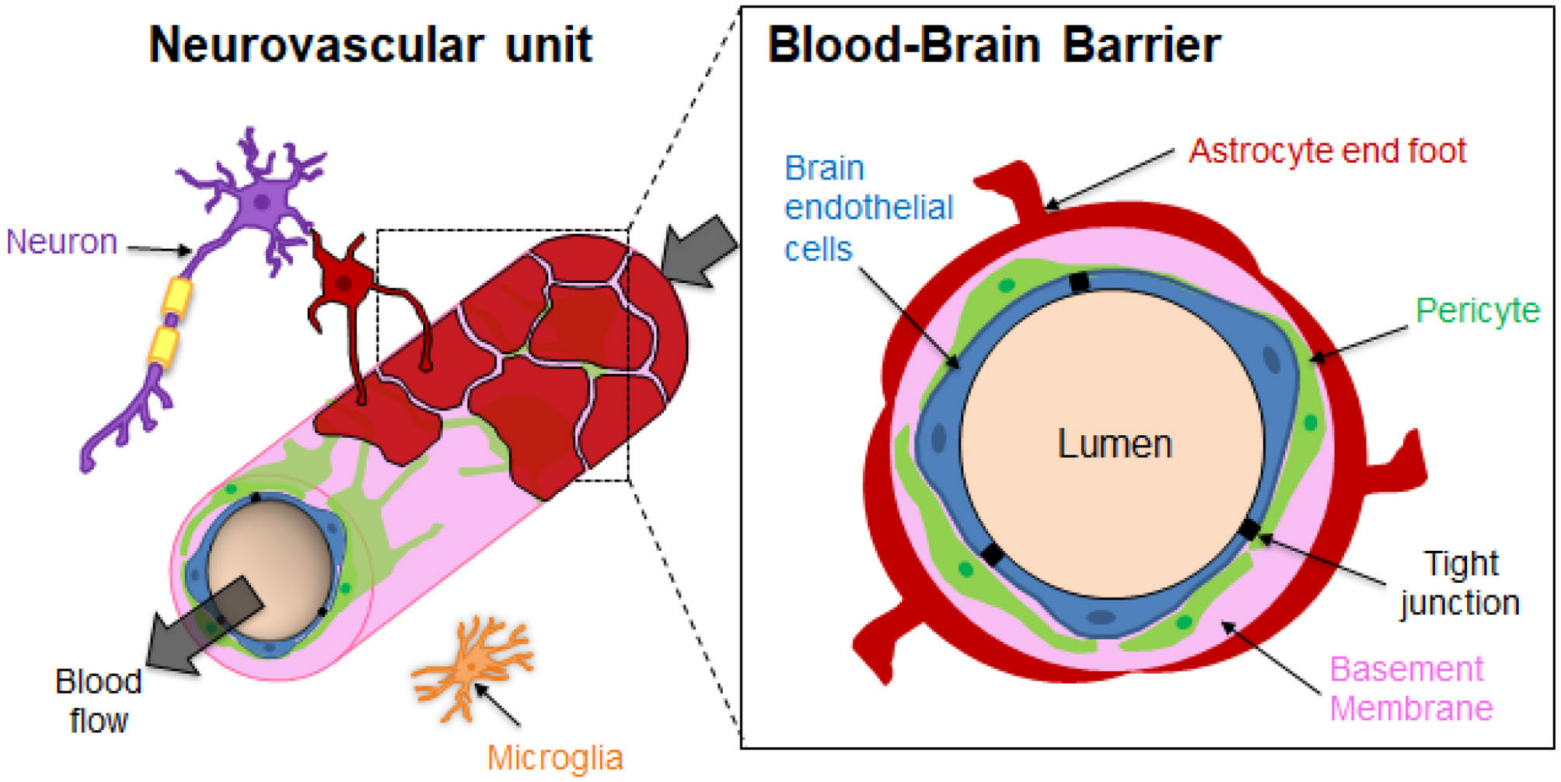
Structural organization of the Neurovascular unit (NVU) and the Blood-Brain Barrier (BBB). The neurovascular unit (NVU) is a structural and functional complex comprising cellular and extracellular matrix components. The NVU include neurons, microglial cells, and the BBB endothelial cells (ECs), pericytes (PCs) and astrocytes (ACs). The components of the NVU interact with each other in a synergistically manner to regulate exchanges between the blood vessels and the brain.

The brain microvasculature's unique organization imparts the restrictive function of the BBB (Figure 1). Specialized non-fenestrated endothelial cells (ECs) are surrounded by pericytes (PCs) and astrocytes (ACs) (Abbott, 2013; O'Brown et al., 2018) themselves ensheathed in the extracellular matrix (ECM). The brain ECM accounts for more than 20% of the total brain volume and is mainly formed by two basement membranes (BMs), namely the endothelial BM and the parenchymal BM (Xu et al., 2019). The BM is mainly composed of laminins, type IV collagen isoforms, nidogen and heparan sulfate proteoglycans (perlecan, agrin), which are mainly directly synthesized by the vascular cells. The BM has a thickness of around 20–200 nm (Timpl, 1989; Engelhardt and Sorokin, 2009; Thomsen et al., 2017b). It should be noted that regional differences of brain BM thickness and expression of BM components such as laminins have been recently observed in mice (Hannocks et al., 2018). The BM provides physical support and transduces cellular signaling events occurring within the BBB (Thomsen et al., 2017a). The brain endothelium greatly differs from that in the rest of the human body since the ECs are highly polarized, display minimal vesicular trafficking and have high expression of tight junction (TJ) proteins and transporters (Chow and Gu, 2015). The TJs between the brain ECs cells lead to high endothelial electrical resistance and low paracellular permeability. The transendothelial electrical resistance (TEER) reflects the junction development and barrier integrity. Although TEER values across human brain endothelium cannot be measured *in vivo*, TEER measurements performed on rat and frog brains have been estimated to be around 1,500–2,000 Ω.cm^2^, much higher than the 3–33 Ω.cm^2^ in the other tissues (Stamatovic et al., 2008). Although ECs are the main contributor of the BBB's properties, some studies have also emphasized the important role of PCs and ACs in the brain vasculature induction and maintenance (Shepro and Morel, 1993; Alvarez et al., 2013). PCs enhance the angiogenesis; control the vaso-regulation owing to their contractile properties, as well as the development and maintenance of integrity and function of blood vessels. Brain vessels have the highest pericyte coverage in the body, with an EC:PC ratio between 1:1 and 3:1 (Shepro and Morel, 1993). The pericyte density can be positively correlated with a decrease in the vascular permeability. It has been demonstrated that pericytes regulate the molecular trafficking into the brain by enhancing the formation of TJs from the ECs (Kutcher and Herman, 2009; Daneman et al., 2010; van Dijk et al., 2015). The astrocyte end-feet processes cover about 90–98% of the brain vasculature (Pardridge, 2007). However, a recent study by Korogod *et al*. suggested that percentage of astrocytic coverage of brain capillaries from mice cerebral cortex was significantly lower than previously observed (about 62%), compared to the 94% coverage obtained with the conventional chemical fixation method (Korogod et al., 2015; Abbott et al., 2018). Astrocytes secrete factors that upregulate the expression of TJs of the ECs, transporters and specialized enzyme systems (Janzer and Raff, 1987; Abbott et al., 2006; Daneman and Prat, 2015). ECs, ACs and PCs ultimately form a dynamic functioning structure with microglial cells and neurons, known as the neurovascular unit (NVU), that tissue engineering aims to reproduce in the laboratory (Abbott et al., 2010; Bell et al., 2020).

In this review, we focus on the latest trends in terms of 3D *in vitro* modeling of the brain vasculature in both healthy and tumor contexts. Their potential as a platform for drug screening and toxicological assessments will also be discussed. All key studies' parameters are summarized in Table 1.

**Table 1:** Summary of the key properties of the different healthy and tumor brain microvasculature models.

**Model type**		**Cell types**	**Vessel Lumen**	**Vessels diameter**	**ECM**	**Permeability assay**	**Drug assay**	**Toxicity assessment**	References*
Spheroids	Healthy	Human primary brain ECs, PCs, ACs	/	/	/	/	/	/	Urich et al., 2013
		Human primary ACs, PCs, ECs or ECs cell line (hCMEC/D3)	/	/	/	4.4, 70 and 155 kDa dextran, angiopep-2, cell penetrant peptides	BKM120 (penetrant drug), dabrafenib (non /penetrant drug)	PgP inhibitor	Cho et al. (2017)
		Human brain ECs, PCs, ACs, oligodendrocytes, microglia, and neurons	/	/	/	Albumin, Immunoglobulin G	Secoisolariciresinol diglucoside, 2-Arachidonyl glycerol	Hypoxia, mercury ions, MPP+, MPTP	Nzou et al., 2018
	GBM	GBM cell line (U87MG) and HUVECs	/	/	/	/	/	/	Avci et al., 2015
ECM-based vasculature	Healthy	Human primary brain ECs, ACs, PCs	/	/	Matrigel®	/	/	/	Shima et al., 2020
		Human ECs, multipotent mesenchymal stromal cells	Yes	14 μm	Gelatin, polyethylene glycol	/	/	/	Klotz et al., 2019
		HiPS derived ECs	/	150 μm	Type I collagen	Lucifer yellow, 10 kDa dextran	/	/	Grifno et al., 2019
	GBM	HUVECs, human GBM cell lines (U87MG, T98, or LN-z308)	No, but sprouts	/	Fibrin	/	/	/	Chen et al., 2009
		HUV ECs, and primary human fibroblasts, U87MG,	Yes	25 μm	Methacrylamide-functionalized gelatin	/	/	/	Ngo and Harley, 2017
Bioprinted model	GBM	HUVECs, human lung fibroblasts, U87MG	Yes	10-25 μm	Gelatin, alginate, fibrin	/	Temozolomide, sunitinib, or combination of the two drugs	/	Han et al., 2020
		HUVECs, U87MG, or human primary GBM cells	/	/	Porcine brain dECM or collagen	/	KU-60019, temozolomide, and cisplatin	Chemoradiation,KU-60019, temozolomide, and cisplatin	Yi et al., 2019
		Human patient derived GBM stem cells, macrophages, ACs, and neural stem cells	/	/	Gelatin methacrylate and glycidyl methacrylate-hyaluronic acid	4 kDa dextran	Abiraterone, vemurafenib, and ifosfamide, EGFR inhibitors (erlotinib and gefitinib) and temozolomide	Abiraterone, vemurafenib, and ifosfamide	Tang et al., 2020
Flow-based vasculature	Healthy	HiPS-ECs, human primary PCs and ACs	Yes	25 μm	Fibrin	10 kDa or 40 kDa dextran	/	/	Campisi et al., 2018
		Human ECs, PCs and ACs cell lines	Yes	25 μm	Type I collagen and Fibrin	/	/	/	Figarol et al., 2020b
		ECs cell line, human primary PCs and ACs	/	400 μm	Matrigel®	4 kDa or 40 kDa dextran	/	/	Ahn et al., 2020
		HUVEC, primary human fibroblasts, rat cortical neurons	Yes	50 μm	Fibrin	20 kDa and 70 kDa dextran	/	/	Bang et al., 2017
	GBM	U87MG, HUVEC	Yes	/	Fibrin	70 kDa dextran	/	/	Xiao et al., 2019

*ACs, Astrocytes; dECM, Decellularized extracellular matrix; ECs, Endothelial cells; EGFR, endothelial growth factor receptor; GBM, Glioblastoma; HiPS, Human induced pluripotent stems cells; HUVEC, Human umbilical vein endothelial cell; kDa, Kilodalton; MPP+, 1-methyl-4-phenylpyridinium; MPTP, 1-methyl-4-phenyl-1, 2, 3, 6-tetrahydropyridine; PCs, Pericytes; P-gp, P-glycoprotein; /, No data*.

* *It is by no means an exhaustive list but it gives an indication of the typical examples for each type of model*.

## Modeling the Healthy Brain Microvasculature

### Spheroid Models

**Figure 2:**
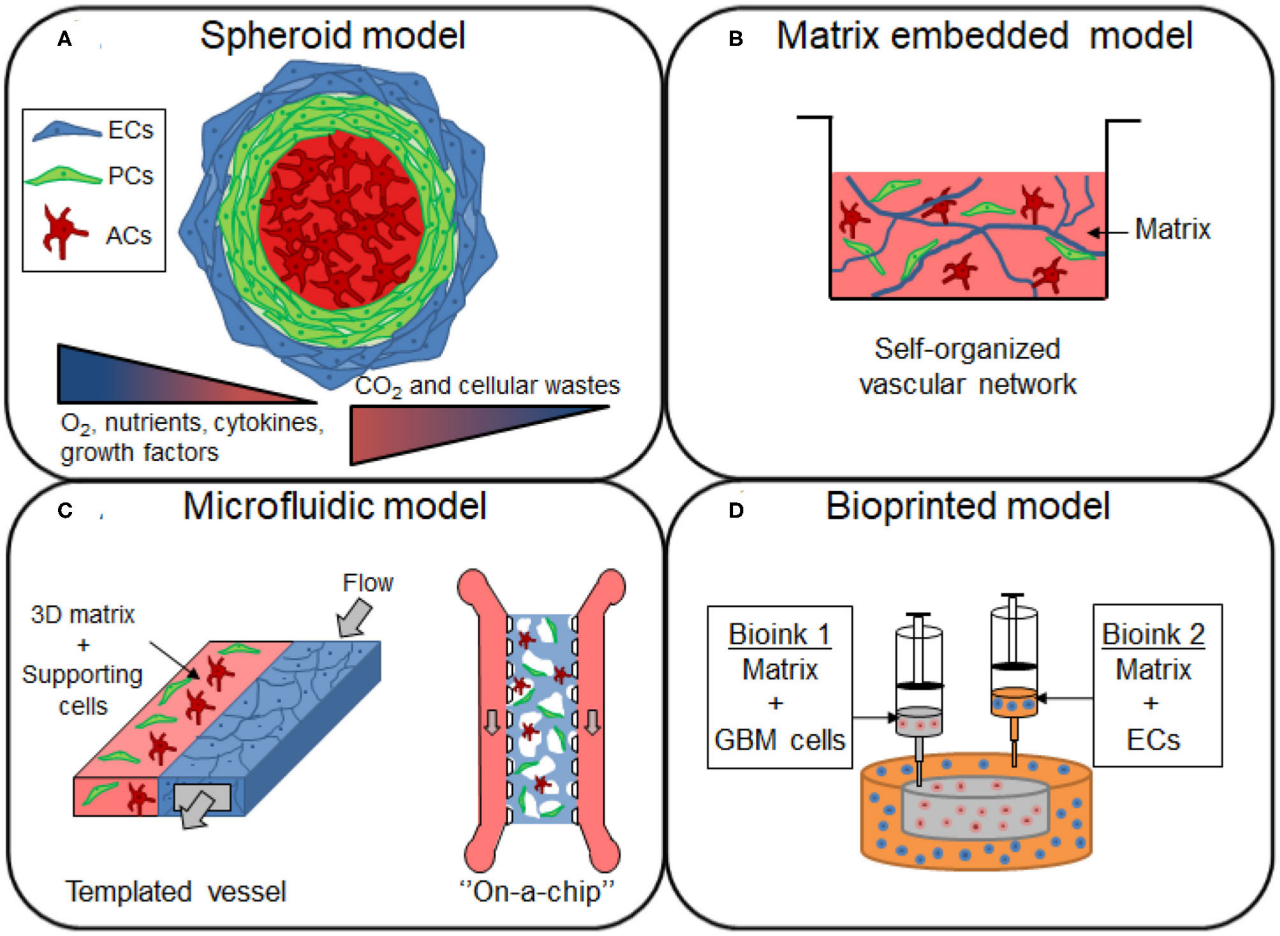
Overview of the different 3D models of brain vasculature. **(A)** Spheroids are formed by the aggregation of single or multiple cell types from the BBB. The cells from the BBB can self-organized within the 3D structure, with endothelial cells (ECs) and pericytes (PCs) localized in the periphery, and with astrocytes (ACs) forming the core of the spheroid. Cells proliferation induce an increase in size of the spheroid, establishing gradients of nutrients, oxygen (O_2_), carbon dioxide (CO_2_) and cellular wastes. **(B)** Cells of the BBB are mixed in a matrix made of native or synthetic materials to provide a scaffold that support cell migration and the formation of a self-organized vascular network, with sometimes the presence of lumen. **(C)** In microfluidic devices, the blood flow is simulated by the injection of the medium inside the channel of the device. The generated shear stress enhances the barrier functions of the blood vessel, restricting the permeability of the molecules. **(D)** Construction of glioblastoma (GBM) model by bioprinting method. A compartmentalized structure with GBM cells in the core and ECs in peripheral regions can be achieved by sequential deposition of various bioink formulations.

Spheroids are 3D spheroidal cellular aggregates that can be prepared either on low-adherence support or by using the hanging drop technique. This model type can be used to study drug transport through the BBB and for developing brain-penetrant drugs for the treatment of neurological diseases (Figure 2A). It presents the advantage of having direct cell-cell interaction, which is lacking in the Transwell® culture. Spheroids could provide a very versatile approach for the HTS of drugs, and show a potential for being conventionally used in pharmaceutical studies (Urich et al., 2013; Cho et al., 2017; Boutin et al., 2018). They can easily be scaled-up due to the relative ease of culture and reproducibility as well as the low cell numbers required (Seo et al., 2020). BBB spheroid models can be obtained by co-culturing ECs, ACs and PCs. Several studies have developed a multicellular BBB spheroid model using the 3D hanging-drop method, ACs in the center of the spheroid surrounded by a layer of PCs, and with brain ECs on the external face of the spheroid (Urich et al., 2013; Cho et al., 2017). In the hanging-drop technique, droplets of cells are suspended from the underside of an adherent tissue culture lid. The cells accumulate at the tip of the drop, driven by the influence of the gravity, which can then spontaneously aggregate and grow into a spheroid. Although this technique is relatively simple and enables a relative uniform spheroid size, it is incompatible with high-throughput, as the culture media should be manually changed. The permeability of BBB spheroids has been found to be comparable to *in vivo* mice studies when cultured in the absence of pro-angiogenic factors in a culture medium such as vascular endothelial growth factor-A (VEGF-A), shown to decrease the expression of TJs like Zonula Occludens-1 (ZO-1) (Cho et al., 2017). Cho et al. proposed a fully human model composed of either immortalized cell line ECs (hCMEC/D3) or primary microvascular ECs, in co-culture with primary ACs and PCs. Brain ECs demonstrated barrier function properties, including tight junctions formation and efflux transporter activity, as assessed by the high expression level of P-gp efflux pump on the surface of the spheroid. In this model, they however use immortalized cell line, hCMEC/D3, which has been shown to have a relatively low TEER (~50 Ω·cm^2^), due to their limited ability to form complete formation of TJs. This spheroid model also failed to accurately correlate the permeability of cell penetrating peptides between *in vitro* human model and *in vivo* mice model. This lack of predictability could potentially result from the model configuration or from the species differences between both models. The spheroid technique could be improved to elaborate more complex human cortical structures. Nzou *et al*. cultured six cell types (brain ECs, PCs, ACs, microglia, oligodendrocytes, and neurons) forming an engineered NVU, which could serve as a platform for neurotoxicity assessment (Nzou et al., 2018). The same research group later demonstrated the effects of hypoxia and neuro-inflammation on their spheroid model (Nzou et al., 2020). They first showed a different profile expression of transport, junctional and BM proteins between normoxic and hypoxic conditions. Hypoxic conditions induced the disruption of the BBB in their model, explained by the upregulation of pro-inflammatory cytokines and chemokines. An increased permeability of 5 kDa dextran and fluorescein labeled immunoglobulin G (IgG) were indeed observed after the treatment of cytokines mix (IL-2, IL-6, VEGF, and TNF-α). Most of the existing models of brain vascular-like spheroids are typically prepared by mixing the endothelial cells with the supporting cells, but a different approach has recently been proposed by Song et al. In their study, vascularization was introduced by the fusion of two types of spheroids derived from human induced pluripotent stem cells (HiPSCs): cortical spheroids and isogenic endothelial spheroids (Song et al., 2019). The advantage of this method is a high control of spatial compartmentalization of cells. It avoids the cell dissociation-re-association processes which usually lead to important cell loss with conventional methods. This model provides an interesting approach for cell-cell interactions, making it valuable for the design of the next generation of spheroids, as neural-vascular interactions are required to reproduce neurological diseases *in vitro* and the testing of drugs that requires a 3D whole brain structure (Song et al., 2019).

The early drawbacks of these BBB or NVU spheroids are the non-physiological geometry of the endothelium. Newly developed techniques have conversely shown the possible organization of a capillary-like network, including lumen within the spheroids, using primary-derived postnatal rodent cortex as a cell source and a low adherence coating made of agarose. The cells can secrete themselves proteins of the BM, and the system achieved a cell density and stiffness close to *in vivo* conditions (Boutin et al., 2018). Ulrich et al. proposed a tri-culture spheroid model using human primary ECs, ACs, and PCs in which the cells spontaneously self-organize in a complex arrangement. In this study, a necrotic core was however formed due to the relatively large size (300 μm diameter) and high cell density in the spheroids (Urich et al., 2013). The capillary network, if present, remained scarce and on the surface, thus could not ensure deep penetration of nutrients and oxygenation in the inner part of the spheroids. Some studies have also shown incomplete coverage of ECs that allowed the passage of FITC-IgG. This was due to the variable cellular composition and organization obtained after their self-assembly, making it difficult to consider long-term assessment of drugs (Nzou et al., 2018). Although some studies have reported that cells could secreted their own ECM in the spheroid models (Glimelius et al., 1988; Boutin et al., 2018; Simão et al., 2018), there is, in general, an important lack of ECM to provide a proper cell 3D conformation support and cell signaling transduction. Deficiencies in the organization of a functional vascular network are also found. Although vascularization of such model is not a goal *per se*, it would be another use of the spheroid technics for the modeling of the BBB. Incorporating perfusable vessels in this model type could indeed open up new possibilities for a long-term culture, currently difficult with the non-perfused spheroid model (Nashimoto et al., 2017). Transport studies in such models would need to overcome challenges such as lumen access and quantification of the transported compounds. Although spheroids have been extensively applied for drug testing studies, they cannot yet be used for drug discovery purposes. Analyses performed on BBB spheroids are an important limiting point for HTS applications. Due to the need for a large number of cells within a test sample, molecular biological assays (western blotting, RT-PCR) are difficult to perform on lysates obtained from spheroids. Several samples have to be pooled to reach sufficient amount needed for both molecular analyses or the detection of non-fluorescent (i.e., drugs) substances by analytical chemistry methods such as High-performance liquid chromatography (HPLC) (Neuhaus, 2020). The difficulty to prepare a large number of spheroids with a homogeneous size also greatly hinders the quantitative analysis of cellular uptake of compounds within the 3D spheroids. The high variability of spheroid size resulting from the use of the conventional methods to prepare spheroids is also a major issue. The size is indeed a crucial factor to consider because it can affect the transport kinetics, the diffusivity and toxicity effects of the tested compounds. Microfluidic platforms have been an emerging approach to prepare uniformly-sized spheroids and simplify liquid handling procedures. Various sizes of spheroids could be generated on a single microfluidic spheroid array by using different well shapes and dimensions (Eilenberger et al., 2021). High-sized BBB spheroids could be prepared with reproducibility, and with diameters ranging from 150 to 450 μm. The impact of the size on the permeation of two chemotherapy drugs, doxorubicin and cisplatin, could be easily investigated. The lack of isolated compartment makes it however difficult to perform analysis independently for each sample. For now, current works have demonstrated the ability to prepare multicellular models, but their assembly into biomimetic structures is still a major challenge to obtain more relevant models.

### Matrix Embedded Models Using Extracellular Matrix or Synthetic Materials

The incorporation of a 3D matrix is important in vascular and neural tissue engineering as it provides support and comprises appropriate ECM factors that enable the recapitulation of the morphological and functional characteristics of those cells (Placone et al., 2015) (Figure 2B). For a more relevant 3D microenvironment, the choice of material is a key factor to take into account when designing the cell scaffold. Neuronal or microvascular cells can be embedded in biocompatible materials obtained from synthetic or natural sources (Willerth and Sakiyama-Elbert, 2007).

Synthetic materials possess many advantages that make them attractive for tissue engineering, such as the possibility to finely tune the mechanical properties and degradation rates by modifying the crosslinking degree or the composition. The porosity can be modulated by adjusting polymer chain lengths and density for supporting cell migration and vasculogenesis processes. Biocompatible synthetic materials used for modeling brain microvasculature include copolymers such as poly(ethyl acrylate), poly(hydroxyethyl acrylate), poly(methacrylic acid) (Veiga et al., 2011), or polyethylene glycol (Barry et al., 2017), which can be further modified with binding peptide sequences, such as arginylglycylaspartic acid (RGD), for cell adhesion.

Natural materials can also be used because they are inherently bioactive, contain endogenous binding motifs for cell adhesion and cell infiltration, and also exhibit similar mechanical properties as the soft brain tissues. Such materials include type I collagen matrix (De Jong et al., 2018; Ruano-Salguero and Lee, 2018; Grifno et al., 2019; Figarol et al., 2020a), decellularized ECM (dECM) (Gao et al., 2017; Praça et al., 2019) and Matrigel® (Feng et al., 2011; Patel and Alahmad, 2016). Type I collagen is present in most human tissues including the brain, even though its concentration is considered to be lower than in other parts of the body (Ferro et al., 2020). Although it is known that collagens are not abundant in the brain ECM and are only limited to the vascular BM (Novak and Kaye, 2000), type I collagen remains the most extensively used scaffold due to its easy access and mechanical tunability. Hydrogel made from type I collagen microfibers allowed to reach a stiffness and a Young's modulus close to mammalian brains (Grifno et al., 2019; Figarol et al., 2020a). Conversely, type IV collagen has relatively low mechanical properties (Birk and Silver, 1987), making it difficult to be applied alone for 3D brain vasculature modeling. Matrigel® is a soluble and sterile extract of BM from a mouse sarcoma tumor (Engelbreth–Holm–Swarm), which is characterized by a high content of ECM proteins. However, its highly variable composition and stiffness among production lots limit its potential use (Klotz et al., 2019). Decellularized ECM, characterized by a removal of native cells, can represent a good alternative for maintaining native tissue ECM components in terms of protein composition and growth factors as well as physical properties (Gao et al., 2017). Brain dECM is however usually not from human source but usually from porcine or rodent origins (Sood et al., 2016; Yi et al., 2019), which can potentially affect the relevance of the BBB model. Shima *et al*. recently developed a new BBB model in a culture insert where the membrane was made of thin collagen Vitrigel® (Shima et al., 2020). First reported by Takezawa *et al*., collagen Vitrigel® is a novel biomaterial obtained by a vitrification process, providing a new scaffold for the construction of 3D cultures (Takezawa et al., 2004). Due to its highly dense network of collagen fibrils, it can be a good equivalent to *in vivo* membrane. For example, it can be used to recapitulate more closely the physiological cellular organization of the BBB as compared to the traditional culture insert models (or Transwell®) where a synthetic polyester membrane creates a separation between cells (Shima et al., 2020). The brain cells embedded in Matrigel® can have direct cell-cell and cell-ECM interactions. Moreover, this model has the advantage of easy isolation of the tissue after the experiment for further characterizations by simply using acetone to detach the collagen Vitrigel® membrane.

Recent studies have tried to obtain a more complex environment by creating composite biomaterials including collagen, dECM and Matrigel® (Sood et al., 2016), or hyaluronic acid (HA), collagen and Matrigel® (Placone et al., 2015). Modeling an appropriate basement membrane is crucial not only for the maintenance of BBB integrity, but also because it undergoes various modifications that are still not well understood during neurological pathologies (Thomsen et al., 2017a). HA, for example, is involved in vasculogenesis processes through the regulation of cell proliferation and migration (Hanjaya-Putra et al., 2012). An acrylated HA hydrogel modified with RGD peptide and containing MMP-sensitive crosslinker enabled a spatial control of the vasculogenesis of ECs under ultraviolet exposure of the hydrogel (Hanjaya-Putra et al., 2012). Although adding ECM components artificially can be an interesting approach, BBB cells also form their own ECM over the time, which may be in term more physiologically relevant. The ECM origin and composition should be carefully considered to ensure the accurate replication of the brain vasculature *in vitro*. Further work should focus on increasing the complexity of ECM composition and the replication of the native BM, which is involved in several processes, including cell differentiation, homeostasis, tissue maintenance, and cell structural support. However, it is still unknown whether any existing biomaterial can simultaneously support neurovascular assembly or if novel composite materials will be necessary to address this issue.

### Microfluidic Models

In physiological conditions, the blood flow has mechanotransductive effects on ECs, known to be of great importance for cell differentiation and TJ formation (Cucullo et al., 2013; Raasch et al., 2016). Indeed, shear stress developed by blood flow can increase TJ proteins, for example ZO-1 expression and reduces the permeability properties (Cucullo et al., 2013). Therefore, more advanced BBB models have been developed using different materials, designs, and strategies, allowing for media to flow, hence generating a shear stress (Figure 2C).

Dynamic culture relies on the construction of templated vessels to mimic the blood vessel structure, which can be made of rigid channels or by using ECM components. The blood flow is simulated using a pulsatile pump which injects the medium inside the blood vessel and reproduces the rheological features like those observed *in vivo*. The flow can also be driven by gravity and by capillary effect to eliminate the need for pumps for a more robust and scalable model (Sugihara et al., 2020; Yu et al., 2020). ECM channels can display an array of blood vessels embedded in a hydrogel, usually type I collagen (Kim et al., 2015; Partyka et al., 2017; Yu et al., 2020). The preparation of the microchannels often involves the use of microneedles to create holes in the matrix, resulting in vessels with a diameter of around 300 to 400 μm (Kim et al., 2015; Yu et al., 2020). Frequent collapses of those microchannels follow the withdrawal of these microneedles. Partyka et al. recently obtained channels with a diameter size ranging from 180 to 220 μm (Partyka et al., 2017). They demonstrated a higher TEER value when the ECs channel was exposed to a shear stress of 0.5 dyn.cm^−2^, compared to the static conditions (Partyka et al., 2017). These models still have however relatively large diameters compared to the dimension of human brain microvasculature *in vivo* [arterioles and venules 10–90 μm; capillaries 7–10 μm (Campisi et al., 2018)]. The development of microvessels with diameters of 7 to 10 μm is a goal that indeed still challenging in tissue engineering. Progress could also be made regarding the brain microvasculature morphology and development in terms of mature cell-cell interactions, as well as physiological blood flow rates and wall shear stresses, necessary to stimulate mechanosensing and mechanotransduction pathways, being still far from a realistic representation of transport exchange mechanisms at the level of brain capillaries (Campisi et al., 2018).

Other devices, termed “on-a-chip” can be used and rely on microfluidic devices. An endothelium monolayer is, in most cases, seeded on the inner walls of rigid channels often made of polymers such as polydimethylsiloxane or polypropylene. These walls are pre-coated with adhesive proteins such as fibronectin to facilitate ECs adhesion. ECs are allowed to adhere under static conditions during a short period of time (usually 24 h) before flow perfusion. The devices generally contain at least two compartments: one with these ECs, and one with supporting cells separated by a porous membrane (Booth and Kim, 2012; Kim et al., 2016; Park et al., 2019). The membrane enables the shear flow to be applied to both compartments distinctly (Raasch et al., 2016). However, in these models no direct cell-cell contact can be achieved between the ECs seeded and ACs and PCs, limiting the cell interactions to paracrine exchanges solely. Some studies have incorporated an ECM component when seeding cells in the microchannel during the flow culture, and proved its importance in promoting the formation of the vessels lumen (Kim et al., 2016; Lee et al., 2020). Very few teams have yet managed to obtain a central channel with the three cell types of the BBB organized in 3D with direct cell-cell and cell-ECM interactions. Although medium flow experiments have only been carried out from a few hours (Campisi et al., 2018) to a few days (Figarol et al., 2020b) in such systems, it still seems to be a path for obtaining a proper self-organized microvascular network.

The average shear stress is estimated to range from 4 to 30 dyne.cm^−2^ within the arterial circulation, and from 1 to 4 dyne.cm^−2^ in the veins (Wong et al., 2013). However, the shear stress applied to the cells greatly depends on the diameter of the vessels and the brain area. For capillaries of 10 μm diameter, the calculated shear stress is about 10 to 20 dynes.cm^−2^, which corresponds to a flow rate of about 6 to 12 nL.min^−1^ (Kamiya et al., 1984; Wong et al., 2013). Flow rates in the current microfluidic models vary from 1.3 μL.min^−1^ (Cucullo et al., 2013) to 370 μL.min^−1^ (Figarol et al., 2020b), and are therefore much higher than the values found *in vivo*. These flow rates are relatively high considering the small size of the vessels (around 25 μm diameter) (Figarol et al., 2020b) in these microfluidic models compared to the physiological values found in the human brain capillaries. The brain vasculature models with this high laminar shear stress have nevertheless demonstrated a lower permeability to templated tracer molecules (i.e., sucrose) compared to static models (Santaguida et al., 2006). This attests the significant role that laminar shear stress plays in enhancing the expression of TJ protein and stimulating a stable BBB phenotype. Despite their advantages and potential development pathways, microfluidic platforms are still not widely used for toxicity screening due to the difficulty of handling, and the special and sometimes expensive equipment required. Moreover, as for now, the number of samples per device is still limited, making the translation to high-throughput screening (HTS) laborious.

Several research projects focusing on the development of flow technologies are now emerging in Asian countries for safety and pharmacokinetic evaluations in the process of drug discovery (Tissue Chip Initiatives and Projects, 2015; Hong et al., 2017; Ahn et al., 2020; Lee et al., 2020)^1^. For example, the Japan Agency for Medical Research and Development (AMED) initiated a project named the “Japan Regenerative Medicine Project” aiming to develop micro-physiological systems using HiPSC or other stem cells seeded in microchips reproducing several organs of the human body, with a focus on the brain microvasculature^1^. Overall, microfluidics is now becoming a fast-growing area of research to facilitate toxicity assessment, either on models of isolated organs, or in the long term, on several miniaturized human organs connected to address organ-to-organ interactions (“human-on-a-chip” projects) (Sung et al., 2019).

## Modeling Vascularized GBM

Mimicking the physiochemical properties of the BBB is particularly interesting to understand its involvement in various neurological diseases including GBM (Jorfi et al., 2018). Incorporating cancer cells in models of the healthy brain vasculature is a useful first step to understanding the BBB dysfunction that may occur under pathological conditions.

### GBM Spheroid Models

The 3D spheroid model is an interesting strategy for the construction of 3D tumor models as it recapitulates cell-cell and cell-ECM interactions between tumor cells and the surrounding tissue microenvironment (Avci et al., 2015). This model reproduces numerous structural, physiological and biological characteristics found *in vivo*, such as nutrient and oxygen gradients (Nunes et al., 2019). Hypoxic conditions and acidosis were shown to be major drivers of cancer progression, since they stimulate the production of anti- and proangiogenic factors, resulting in neovascularization and chemotherapy resistance (Kolenda et al., 2011; Hardee and Zagzag, 2012). As stated in the section Spheroid Models, using spheroids model faces some limitations in the reproduction of a healthy brain microvasculature (Urich et al., 2013). Conversely, the technic and shape can properly reproduce tumors with a necrotic and hypoxic core, and a peripheral area with higher oxygen levels and proliferation rates. GBM spheroids are often prepared using patient-derived cells for a better representation of the heterogeneity of GBM population (Avci et al., 2015; Quereda et al., 2018; McCoy et al., 2019). The main limitations of these models remain the lack of ECM and vascularization. It is now well-established that the tumor microenvironment (TME), hence the ECM, plays a biophysical role in controlling tumor growth and spreading. Recent work by McCoy et al. has proposed a model of a GBM spheroid in distanced co-culture with ECs (McCoy et al., 2019). GBM spheroids were embedded in a collagen gel in the top chamber of the Transwell® insert while ECs were seeded on the coverslip in the well-bottom. They aimed to study the interactions between both cells and the influence of the vascular network on GBM invasion. They first wanted to determine if Interleukin-8 (IL-8) secreted by ECs could alone induce an increase of GBM tumor cell invasion in the 3D co-culture without any contact between GBM cells and ECs. Interestingly, it was found brain ECs stimulated GBM tumor cells migration through the membrane insert, potentially explained by the enhancement of the stem-like behavior of the migrated GBM population, suggested by the increase of the nestin expression. ECs were then seeded into the collagen hydrogel surrounding GBM spheroids for direct cell-cell contact. Interestingly, a synergistic crosstalk was demonstrated between ECs and GBM cells, as IL-8 secreted by ECs could stimulate the invasion of GBM cells inside the collagen hydrogel and the growth of the spheroid. Reciprocally, GBM cells stimulated vessels sprouting toward the tumor site. The results of this study emphasize the importance of the interplay between ECs and GBM and the presence of the vascular component for the design of an appropriate tumor model. More recently, higher reproducibility of the models was also achieved thanks to a microfluidic-based approach which promotes spheroid formation with uniform size (Ohnishi et al., 2014; Suryaprakash et al., 2019).

### GBM Matrix Embedded Models

GBM is characterized by its highly invasive and infiltrative capacities. At a later stage of the tumor development, cancer cells spread in surrounding brain parenchyma through GBM-driven angiogenesis and neovascularization processes (Hardee and Zagzag, 2012).

Despite the fact that biologically sourced biomaterials offer a rich environment for studying GBM invasion *in vitro*, they present some limitations in the modulation of their mechanical properties and chemical structures. For example, ligand density, stiffness, and porosity cannot be easily changed, enabling only a partial mimicry of tumor characteristics. Thus, synthetic material are often preferred as cell scaffolds because it is easier to tune the hydrogel stiffness, an important modulator of the morphology, proliferation, and motility abilities of GBM cells (Ananthanarayanan et al., 2011; Wang et al., 2014; Ngo and Harley, 2017). Current platforms usually use GBM cancer cells in monoculture in 3D scaffolds made of gelatin (Heffernan et al., 2015), chitosan (Florczyk et al., 2013; Wang et al., 2016), chitosan-alginate (Kievit et al., 2010), type I collagen (Rao et al., 2013), Matrigel® (Jin et al., 2009), or polyethylene glycol (Pedron et al., 2013; Wang et al., 2014; Avci et al., 2015). Recently, Kou *et al*. used patient tissue-derived dECM to evaluate GBM mobility (Koh et al., 2018). Interestingly, the morphology and dynamics of invasion of tumor cells were different compared to type I collagen hydrogel. Many of the current models tend to incorporate HA in synthetic scaffolds to provide a more physiological and predictive model. HA is not only a major component of the brain ECM, but is also secreted by GBM cells (Jin et al., 2009; Wang et al., 2014; Ngo and Harley, 2017). Malignant cells can interact with HA by receptors such as CD44, which promotes the invasive and infiltrative phenotype of GBM cells in the ECM (Heffernan et al., 2015). So far, only a few studies have managed to vascularize their GBM models. Some studies have however co-cultivated ECs with GBM cancers cells, and have started research on the evaluation of GBM behavior in the perivascular niche (Chen et al., 2009; Nguyen et al., 2016; Ngo and Harley, 2017). U87-MG GBM cell line was co-cultured along with HUVECs and human fibroblasts onto a methacrylamide functionalized gelatin hydrogel (Ngo and Harley, 2017). Interestingly, U87-MG cells were mainly found localized within 40 μm of an endothelial cord. After 14 days culture, a regression of the ECs network was observed, as evidenced by a reduction of the vessel branching number, vessel length and junction number. The degree of disruption was positively correlated with the density of GBM cells incorporated in the hydrogel. It is now widely accepted that tumor blood vessels differ from healthy vessels in terms of structure, permeability, and basement membrane deposition (Ngo and Harley, 2017), even if recent studies suggested that the permeability of the vasculature may remain intact in several brain tumor regions (Sarkaria et al., 2018). Unfortunately, the current studies that managed to prepare a 3D *in vitro* model of GBM with ECs did not use cells with neural origins (Ngo and Harley, 2017), or still lacked an appropriate 3D conformation of the microvessels in a lumenized vessel network, which is a critical step in forming mature blood vessels (Chen et al., 2009; Nguyen et al., 2016).

### Bioprinted Tumor GBM Model

ECM-based tumor models have their own constraints, such as the limited control over the tumor cell distribution within the hydrogel. Bio-printing can solve this issue under physiologically relevant conditions using a cost-effective approach with high reproducibility, which is highly desired for HTS (Parra-Cantu et al., 2020) (Figure 2D). Several studies have reported models with 3D bioprinted GBM cancer cells alone (Lee et al., 2020), or in co-culture with HAs, neural cells (Tang et al., 2020), or even macrophages (Heinrich et al., 2019; Tang et al., 2020). These latter studies are of relevance since GBM is characterized by a high infiltration of macrophages and microglia populations in the tumor site.

ECM-based materials have been used for the preparation of bioinks, for example fibrin (Lee et al., 2019; Smits et al., 2020) or methacrylated gelatin (Heinrich et al., 2019). After printing or while printing, they are polymerized and offer a stiffness-controlled support for the 3D conformation of GBM cells. Heinrich et al. fabricated a 3D bioprinted model, that included both GBM cells and macrophages in a geometrically sophisticated “mini-brain” (Heinrich et al., 2019). They could specifically study the crosstalk of both cell types. They found that GBM cells were able to recruit macrophages to provide support for their own survival and proliferation. Han et al. prepared a vascularized GBM by printing ECs on which were placed GBM spheroids (Han et al., 2020). This model yielded a large open vascularized tissue with diameters ranging from 10 to 25 μm after 7 days of culture. The microvessels vascularized the tumor spheroid and enhanced the migratory phenotype of GBM cells. Yi et al. developed a bioprinted GBM on-a-chip supported by a brain-derived ECM using patient-derived GBM cancer cells in co-culture with Human Umbilical Vein Endothelial Cells (HUVECs), with each cell type compartmentalized in specific regions of the model (Yi et al., 2019). Upregulation of SOX2 and NES genes cells was only found when cells were separately printed and with the oxygen gradient, indicating the promotion of a resistance phenotype of cancer cells under such conditions (Yi et al., 2019). These last two models showed potential application as drug screening platforms due to their fast and easy production and the controllable size of the vascularized tumor tissue. Despite the promising results, they will require further improvements to better recapitulate the glioblastoma microenvironment. The current models of vascularized GBM mainly rely on the use HUVEC as a source of ECs for the formation of lumenized vascular network (Yi et al., 2019; Han et al., 2020). HUVECs should be replaced by brain ECs, as they lack tight junction properties necessary to reproduce more adequately the barrier functions of the brain vasculature (Uwamori et al., 2019). Other relevant neurovascular cell types should also be added such as human ACs, PCs or microglia, as they can potentially modify drug efficiency outcomes (Heinrich et al., 2019; Han et al., 2020). The differences in tissue stiffness compared to normal tissue should be also investigated, since it can affect the phenotype of cancer cells, and their chemotherapy resistance.

### GBM Microfluidic Models

As stated in the section Microfluidic Models, microfluidic devices enable the *in vitro* reproduction of the blood flow through the brain vasculature. For HTS assays, a few microfluidic models have been designed with ECs and GBM tumor cells, and sometimes other supporting cells (Jeon et al., 2015; Oh et al., 2017; Chi et al., 2020).

The microfluidic tumor–microvascular models focus on the study of immune cell transmigration, metastatic cancer intra- and extravasation processes, as well as tumor-vessel formation (Jeon et al., 2015). Between the different microchannels of a microfluidic system, it is possible to have gradients of biochemical factors or oxygen (depending on whether or not they are perfused). Microfluidics are also particularly pertinent for the study of angiogenesis and vasculogenesis processes within the TME (Zervantonakis et al., 2012; Ma et al., 2018), and allow a better understanding of the tumor spreading (Tsai et al., 2017). As previously stated, the TME is characterized by the pronounced hypoxia in the tumor core. This induces metabolic changes in the peri-necrotic niche, and subsequent neo-vascularization process and invasive phenotypes (Chen et al., 2009; Tsai et al., 2017). The specific TME in the proximity of the vasculature could moreover favor glioma stem cells (GSCs) proliferation, contributing to the tumor growth and high recurrence of the GBM (Calabrese et al., 2007).

Patient-derived GSCs have been recently used for the modeling of vascularized GBM (Liu et al., 2017; Truong et al., 2019). For example, Truong *et al*. constructed a distanced co-culture with HUVEC and GSCs separated by a stromal hydrogel. The migration assay showed that the presence of HUVEC enhanced the migration of GSCs in the direction of the vascular network created by HUVEC (Truong et al., 2019). Although GBM-microvasculature on a microchip is emerging, they are still not totally biologically relevant as they use HUVEC (Liu et al., 2017; Truong et al., 2019). On the other hand, more complex vascularized models of other cancers have already been developed: fibro sarcoma (Zervantonakis et al., 2012), colorectal cancer (Phan et al., 2017) or breast cancer for example (Jeon et al., 2015; Chen et al., 2016; Tsai et al., 2017; Nashimoto et al., 2020). These microfluidic systems are expected to contribute to the development of the next generation of *in vitro* microfluidic GBM models. GBM indeed shares common features with other solid tumors, such as the high invasion of the peripheral tissue. Although GBM metastasis to other organs is very rare, metastases of other cancers can be found in the brain, and their treatment could profit from innovative mixed-tissue models (Xiao et al., 2019).

## Applications of Healthy and Pathological Brain Vasculature Models for Drug and Toxicological Assays

### Selection of the Most Appropriate Model for Drug and Toxicity Screening

The species and cell source are important points to consider for clinical translation of engineered 3D brain microvascular network models. Ideal BBB models are expected to be made of solely human cells in order to meet the translatability requirements. For now, several BBB models however use a combination of human and animal cells. Primary human-derived cells are often a better choice than immortalized cell lines due to their differentiation stages and gene and protein expression levels being close to *in vivo* conditions (Unger et al., 2002; Tsuji et al., 2010). Even if primary human brain ECs can retain some phenotypic characteristics of BBB endothelium, these cells require especially invasive surgeries to be harvested and only a limited cell number can be collected. They are also subject to dedifferentiation and senescence after a few number of passages when cultured *in vitro* (Weksler et al., 2005). Their use is thus difficult for the preparation of HTS platform. Alternatively, many BBB models were constructed using brain cells from rodent or porcine origins (Adriani et al., 2017; Bang et al., 2017; Boutin et al., 2018). Brain animal ECs have indeed demonstrated enhanced barrier functions, such as higher TEER, as compared to the human immortalized cells (Helms et al., 2016). Although co-culturing with cells from different species remains an attractive approach due to the easy access of cells, it is however highly desirable to achieve a fully human BBB model, especially for functional studies (Neuhaus, 2020). A fully human model could be a more appropriate strategy to address the concern raised by the cross-species compatibility and for a better relevance regarding human physiology.

HiPSCs stand as an interesting compromise for the obtaining of neural cells or brain microvascular ECs in larger quantities. HiPS-derived ECs indeed exhibit significantly more biomimetic features than immortalized cell lines. For example, they demonstrate lower paracellular transport properties due to their high TEER values (>1,500 Ω.cm^2^) (Park et al., 2019). They could allow the construction of patient-customized models for future applications in personalized medicine. However, there is a lack of information regarding long-term stability of HiPS-ECs, such as the maintenance of integrity and selective properties of the BBB, crucial for drug screening purposes (Grifno et al., 2019). The development of stable and relevant HiPS-derived ACs and PCs also remains a challenge (Delsing et al., 2020). Obtaining HiPS-PCs is limited by the lack of precise knowledge of the features of brain PCs. Reaching a consensus about their exact origin, their specific markers and functional characteristics would greatly help the development of a standardized protocol to obtain HiPS-PCs. Current protocols for the differentiation of ACs from HiPS are time-consuming and cost-extensive, preventing them to be used routinely. The standardization of the characterization and validation of the HiPS-derived brain microvasculature models could widespread the application of the said-models in drug discovery and toxicity screening (Delsing et al., 2020). An alternative option could also be the combination of hiPS-derived ECs with primary ACs or PCs, as proposed in the microfluidic model of Campisi et al., to alleviate the problem of the different differentiation time for obtaining the different hiPS- derived brain cells (Campisi et al., 2018).

As mentioned below, an appropriate selection of the cells to model the brain microvasculature is critical to obtain meaningful results. Knowing the advantages and disadvantages of each type of brain microvascular model also enables more accurate data interpretation. An appropriate choice of *in vitro* model could indeed help reduce time and money to invest before moving to clinical trials. For the investigation of molecular pathways or transporter kinetics, Transwell® systems might be an ideal choice due to their simplicity, scalability and reproducibility. For transport studies, microfluidic systems may be a better option due to the incorporation of shear stress related constraints and impacts on the cells differentiation. For the hit identification and lead discovery phase of the drug discovery process, or toxicological profile, more sensitive *in vitro* models that replicate the majority of the *in vivo* conditions are necessary. Microphysiological systems may be the future of such models (Wevers et al., 2016), provided that their design meets the requirements for HTS, such as the evaluation of several drugs in parallel on distinct 3D brain vasculature models perfused by continuous flow.

### Evaluation of the Transport of Xenobiotics Across the BBB

The formation of the TJ and the selective permeation are important criteria for the successful engineering of the brain microvasculature. Permeation studies with templated fluorescent tracers, such as dextran with different molecular weights and TEER measurement are the common methods for evaluating the BBB permeability (Yu et al., 2020) before testing for xenobiotic transport potency through the BBB. Xenobiotics are compounds, either from natural or synthetic sources which are foreign to the body, thus are not expected to be naturally found in the organism.

2D models and animal models are still considered as the gold standards for drug screening and toxicity assessment. However, studies involving the use of animals are not only becoming more and more regulated by laws to limit them whenever possible, but they also have a poor predictive ability for BBB penetration and drugs responses on humans (Festing and Wilkinson, 2007). Transwell® models are widely used for HTS (Hatherell et al., 2011), either with a simple culture of an ECs monolayer on the permeable membrane in the upper chamber of the culture insert, or more complex co-culture with ACs, PCs, or neurons (Demeuse et al., 2002; Zujovic and Taupin, 2003; Hayashi et al., 2004). The apical-to-basal permeability (P_A/B_) and the basal-to-apical permeability (P_B/A_) can be determined by analyzing the concentration of tested molecules in the lower and upper chamber of the culture insert. The influx transport in both compartments of the Transwell® model can be identified by the measurement of P_A/B_ and P_B/A._The ratio of P_A/B_ and P_B/A_ is a quite good indicator of the efflux transport through the BBB. However, the ECs in Transwell® models can exhibit higher permeability and lower expression of efflux transporters compared to *in vivo* (Berezowski et al., 2004).

The clearance of xenobiotics from the brain is evaluated via the activity of efflux transporters expressed on the luminal side of the brain ECs which can pump out xenobiotics and endogenous molecule from the brain. P-glycoprotein (P-gp) is a 170 kDa ATP-dependent pump known to have good affinity for a broad range of molecules, including lipophilic or amphiphilic molecules such as anticancer drugs (e.g., cyclosporine A, vinblastine) (Löscher and Potschka, 2005; Terasaki and Ohtsuki, 2005). Although P-gp is the most widely investigated efflux pump, there are also other transporters known to contribute to the efflux transport of xenobiotics though the BBB, namely Multi-Drug Resistance Proteins and Breast Cancer Resistance Protein, which are members of the ATP binding cassette transporters (Löscher and Potschka, 2005; Ni et al., 2010). Elucidating the efflux mechanisms of these transporters expressed by the brain ECs would be very helpful for designing peripherally active drugs and for reducing their undesired penetration of the CNS and associated side effects (Tsuji and Tamai, 1997). Investigating the efflux mechanisms through more relevant 3D models would thus be a great asset for pharmaceutical companies, not only to gain knowledge of the BBB physiology, but also to develop CNS drugs with improved biosafety and enhanced delivery into the brain. It was shown that the expression of efflux pumps, for example P-gp, is upregulated in microfluidic models compared to conventional static ones (Prabhakarpandian et al., 2013; Figarol et al., 2020b). The orientation and transport direction of the efflux pumps remain however often difficult to elucidate in microfluidic models.

## Assessing Xenobiotics Toxicity on the Brain Microvasculature

Brain homeostasis is crucial to ensure proper functioning of the CNS. The brain is subject to frequent stress in our daily life. Oxidative stress, exposures to toxic agents, bacterial and viral infections can notably contribute to the impairment of cerebral biochemical functions.

As current works mainly focus on structural and physiological relevance, the biological responses to xenobiotics and toxicological assays have not yet been systematically assessed (Probst et al., 2018). A few studies address the issue of the effects of xenobiotics toward the brain vasculature in 2D culture (Qosa et al., 2016). Although these models are useful, they neglect some important characteristics such as neurovascular interactions, as well as the protective effects of the BBB and ECM against xenobiotics. Simpson et al. recently showed differences in amyloid beta (Aβ) toxicity between a 2D culture and a 3D collagen-based model with a rat pheochromocytoma cell line PC-12 (Simpson et al., 2020), resulting from the incorporation of the collagen scaffold. Some works have managed to evaluate the toxicity on a 3D cultures of neurons, alone (Smirnova et al., 2016), in co-culture with astrocytes (Wevers et al., 2016; Liu et al., 2020) or with other cells of the NVU (Nzou et al., 2018). Tissue-based 3D models could be used to complement toxicological data obtained with conventional 2D models (Cao et al., 2021). Interests are growing on toxicological data collected on 3D complex models including both brain vascular and neuronal components (Koo et al., 2018). As said, these models could give better insights about the substances which can cross the BBB and induce damages to the brain tissue (Tähti et al., 2003).

Emerging BBB microfluidic devices have been used as a platform for studying the potential adverse effects on the microvasculature of several exogenous compounds. For example, the effect of oxidative stress induced by cationic polymeric nanoparticles (Ahn et al., 2020) or organophosphorus nerve agents (Koo et al., 2018) have been assessed. Organophosphates are commonly found in commercially available products such as insecticides or pesticides. Koo et al. demonstrated *in vitro* the detrimental effects of organophosphates on the brain. These compounds can penetrate the BBB and irreversibly inhibit the activity of the enzyme acetylcholinesterase, causing a toxic accumulation of the neurotransmitter acetylcholine in the brain. Repeated exposures to these compounds can, in turn, lead to neurotoxicity and serious brain damage. Additionally, positively charged nanoparticles have been shown to induce the contraction of the brain vasculature by oxidative stress (Ahn et al., 2018). The presence of exogenous compounds may therefore induce damage that weaken the brain vasculature, perturb brain homeostasis and thus potentially favor the appearance or aggravation of neurological diseases.

### Pathophysiological Damages to the Brain Microvasculature

BBB disruption constitutes a common feature of the progression of several neurological diseases, such as ischemic stroke, Alzheimer's (Shin et al., 2019; Coughlin and Kamm, 2020) and Parkinson's diseases (Desai et al., 2007). In most cases, it results from the modification of the expression of TJs, transporters and receptors expressed at the brain ECs surface, and changes in the vascular density. This increased permeability leads to an accumulation of neurotoxic aggregations of specific proteins in the brain and can enhance oxidative stress (Salim, 2017). The cause and effect relationship between oxidative stress and these protein aggregates are still not fully understood. More mechanistic studies and investigation of cell signaling should be conducted, with the help of the newly developed 3D models, to compare the brain vasculature development under both physiological and pathological conditions.

As stated earlier, both healthy and tumor 3D models are progressing toward higher complexity and greater biomimicry. Although the large production of more complex model is challenging, and they could become more and more pertinent in the future for drug screening purposes if the reproducibility issue is addressed in the preparation method. The current models are valuable to understand the evolution of pathologies occurring in the brain. They can help to focus on the impacts on the vascularization, molecular and cellular events associated with a pathology, such as cancer-driven intravasation of the immune system (Couto et al., 2019; Cui et al., 2019) or tumor metastasis (Diao et al., 2019). Vascular remodeling in GBM is still a much debated topic. Some studies lean toward a systematic BBB disruption (Long, 1970; Zhao et al., 2018), while others suggest that the BBB may not be disrupted in all GBM cases and mention the existence of tumor regions with an intact BBB (Sarkaria et al., 2018). The fate of the brain vasculature network remains to be elucidated, with potentially high heterogeneity resulting from the constant remodeling of the ECM by the tumor cells. Monitoring the evolution of tumor vascularization and oxygenation thanks to brain vasculature on-a-chip models could help to better understand the angiogenic process in tumors, and to address the effects of anti-cancer drugs, especially the anti-angiogenic ones on the brain microvasculature (Elice and Rodeghiero, 2012). Specific pathophysiological conditions could potentially change the neurovascular system (NVS) responses to drugs; replicating those in new engineered models would make them stand as a supplementary step in *in vitro* studies to reduce *in vivo* assays (Cao et al., 2021).

### Assessing Drug Toxicity and Side Effects to the Brain Microvasculature

Current drug discovery is primarily driven by the need for the pharmaceutical industry to test drug candidate libraries against potential targets, drug side effects and toxicity to the CNS. Early safety assessment is indeed required by regulatory institutions for drugs approval (Culot et al., 2008). Therefore, huge efforts are devoted to developing complex *in vitro* models to evaluate the passage of brain anti-cancer drugs through the BBB, identifying the underlying molecular mechanisms and analyzing their impacts on the functions of the neurovascular system (Cecchelli et al., 1999).

Vascular endothelial growth factor (VEGF) is the most abundant and important regulator of angiogenesis in various primary brain tumors, including GBM. Zhao et al. recently showed that hypoxic GBM-derived exosomes containing multiple pro-angiogenic factors including VEGF-A, can induce the proliferation of ECs and angiogenesis. VEGF mediates the increased permeability of the BBB model by reducing the expression of TJ proteins, thereby constructing a suitable environment to support tumor nutrition and proliferation (Zhao et al., 2018). Various therapeutic approaches have been designed to target VEGF-mediated angiogenesis, including VEGF blockade, VEGF trap, and suppression of the expression of its receptor (VEGFR). The anti-VEGF treatment Bevacizumab, for example, is a promising anti-angiogenic compound used to reduce the development of the GBM pathological vascularization by depriving the tumoral tissue of oxygen and nutrients. Although, it showed good results in an *in vitro* 2D model (Miranda-Gonçalves et al., 2017), more controversial results were obtained using a more complex tissue model, such as a GBM xenograft derived from patient tumor spheroids injected in mice (Keunen et al., 2011). The latter study suggested that vascular remodeling induced by the anti-VEGF treatment, enhanced the hypoxic TME and aggressively exacerbated tumor invasion into the healthy brain. Another recent study mentioned the possibility that VEGF antibody could not reach the encapsulated VEGF, essential for its neutralization (Ko et al., 2019). As cancer treatment requires targeting multiple pathways and cell types, the multiplication of tissue engineered models should thus allow collecting more meaningful data for the development of novel drugs to treat or manage neurological diseases.

Other type of treatments such antipsychotics, widely used to manage psychoses that occur with schizophrenia and bipolar disorder have adverse effects on the BBB ECs. Four kinds of antipsychotics (chlorpromazine, haloperidol, risperidone and clozapine) were applied on 2D culture of brain ECs at typical therapeutic concentrations for high dosage treatment. All four antipsychotics showed impairment of cells morphology, increased apoptosis, and decreased of the transcytosis of Evans blue on brain ECs (Elmorsy et al., 2014). Better knowledge of dose repeated treatments impacts on the brain microvasculature are also needed. So far, few studies have investigated the BBB integrity after repeated dose treatment (Fabulas-da Costa et al., 2013). *In vitro* drugs screening 3D systems have been reported (Cecchelli et al., 1999; Elmorsy et al., 2014; T. Phan et al., 2017), especially for cell penetrating agents (Cho et al., 2017) or BBB transient permeabilizer D-Mannitol (Figarol et al., 2020a) to validate the feasibility of HTS using their models. There are however still very few *in vitro* models that have compared and have found a good correlation with *in vivo* data (Lundquist et al., 2002; Culot et al., 2008). Modeling the full NVU offers emerging possibilities not only for studying the drug effects on neuronal function (Nzou et al., 2020). For example, Nzou et al. assessed on a NVU model the impacts of hypoxia-induced neuroinflammation on BBB integrity by incubation of pro-inflammatory cytokines mix. The evaluation should of course not be limited to the neuronal population but also involve the vascular system and glial cells to get a full understanding of the brain homeostasis. An upcoming challenge for 3D *in vitro* toxicological assays and drug screenings is the focus on kinetics data. Precision medicine for cancer therapy can only be achieved if these screenings can be performed in a clinically relevant timeframe (≲14 days). Intermediate and long-term studies are however currently greatly lacking in most of the current works. Kinetics data can greatly help to determine the success of *in vivo* studies, predict metabolic stability of drugs and give insights about their intrinsic clearance from the body.

## Conclusion

Brain microvasculature modeling is still particularly challenging in tissue engineering, as it needs to take into account the unique structure of the BBB, the highly specific brain microenvironment, and intercellular communication. The latest 3D *in vitro* models aim to bridge the gap between *in vitro* and *in vivo*. So far, a trade-off is still often made between complexity and the standardization of the preparation process. The ECM composition and the influence of mechanical shear stress on ECs are increasingly being taken into consideration in brain microvascular engineering. Newly developed 3D models could moreover be of great interest to better understand the tumoral impacts on the microvasculature, and design the next-generation of anti-GBM treatments. Although vascularization is now widely recognized to be of importance in GBM modeling, the influence of blood components and blood cell population are mostly neglected. The analysis of the composition of the secretome of GBM could be helpful for a more accurate diagnosis of the disease. Adverse effects of drugs or xenobiotics to the brain vascular system are still largely misunderstood. The toxicological field is just beginning to yield the benefits of 3D engineered *in vitro* models and on the threshold of further extensive studies using these innovative technologies for the improvement of biosafety and bioavailability of novel neuropathology treatments.

## Author Contributions

MP and AF wrote and edited the manuscript. MM edited and oversaw the completion of the review. All authors have seen and approved the manuscript.

## Conflict of Interest

The authors declare that the research was conducted in the absence of any commercial or financial relationships that could be construed as a potential conflict of interest.

## Footnotes

^1^Project Focused on Developing Key Evaluation Technology : Development of Platform Technology for Drug Discovery through Application of Regenerative Medicine | Japan Agency for Medical Research and Development. Available online at: https://www.amed.go.jp/en/program/list/13/01/004.html (accessed November 1, 2020).
